# From Full Day Learning to 30 Minutes a Day: A Descriptive Study of Early Learning During the First COVID-19 Pandemic School Shutdown in Ontario

**DOI:** 10.1007/s10643-021-01304-z

**Published:** 2022-01-16

**Authors:** Natalie Spadafora, Caroline Reid-Westoby, Molly Pottruff, Jade Wang, Magdalena Janus

**Affiliations:** Department of Psychiatry and Behavioural Neurosciences, Offord Centre for Child Studies, McMaster University, McMaster Innovation Park, Suite 201A, 1280 Main St. West, Hamilton, ON L8S 4K1 Canada

**Keywords:** COVID-19 pandemic, School closures, Online learning, Kindergarten

## Abstract

When the COVID-19 pandemic was declared in March 2020, the lives of families all over the world were disrupted. Many adults found themselves working from home while their children were unable to go to school. To better understand the potential impact of these educational disruptions, it is important to establish what learning looked like during the first school shutdown in the spring of 2020, particularly for the youngest learners who may feel the longest lasting impacts from this pandemic. Therefore, the purpose of the current descriptive study was to gather information on how kindergarten teaching and learning occurred during this time, what the biggest barriers were, and what concerns educators had regarding returning in person to the classroom setting. The sample for the current study was 2569 kindergarten educators (97.6% female; 74.2% teachers, 25.8% early childhood educators) in Ontario, Canada. Participants completed a questionnaire consisting of both quantitative scales and qualitative open-ended questions. Educators reported that parents most often contacted them regarding technological issues or how to effectively support their child. The largest barrier to learning was the ability of both parents and educators to balance work, home life, and online learning/teaching. With regards to returning to school, educators were most concerned about the lack of ability of kindergarten aged children to do tasks independently and to follow safety protocols. Our findings highlight unique challenges associated with teaching kindergarten during the pandemic, contributing to our understanding of the learning that occurred in Ontario during the first COVID-19 shutdown.

**Supplementary Information:**

The online version contains supplementary material available at 10.1007/s10643-021-01304-z.

## Introduction

When the COVID-19 pandemic was declared in March 2020, businesses, offices, and schools were shutdown in Canada, with an unknown timeline for reopening. Not only did school-aged children have to adapt to new and uncertain home and family environments (e.g., not being able to visit loved ones), but they were also forced to navigate through a new world of online learning (e.g., Kuhfeld et al., 2020). From March until the end of the 2019/20 school year in June, the schooling in Canada moved from in-person to virtual, remote, electronic platforms, necessitating a rapid adjustment in the curricula and teaching techniques (Sprang & Silman, 2013). School closures which occurred due to the polio pandemic in 1916 (Doyle, 2020) and the H1N1 influenza pandemic in 2009 (Wang et al., 2020), have been shown to have detrimental consequences for students such as educational and economic losses, increased mental illness, decreased social skills, increased food insecurity, as well as put them at increased risk for adverse experiences, such as family violence (Brooks et al., 2020; Viner et al., 2020).

The overall effectiveness of distance learning depends on the child’s ability to study in the home environment (Belot & Webbink, 2010). Children who live in a setting without the necessary resources to enable adequate learning, such as a place to do homework or access to computers and internet, are less likely to benefit from distance learning programs. This may be particularly true for the youngest learners, as they often require adult supervision and support for distance learning. Moreover, young children may be more likely to experience negative educational impacts due to the learning disruptions. Sprang and Silman (2013) found that young learners were more likely to fall behind when their parents had limited capacity to provide adequate learning support, be it physical (e.g., space, access to a computer), time (e.g., parents who are working full-time), emotional (e.g., heightened stress), or literacy (e.g., limited language of instruction skills).

### Teaching in the COVID-19 Pandemic

Not only can distance learning be challenging for students, it also has the potential to be challenging for educators. For instance, research on teachers in Portugal suggested that the pandemic created or increased teaching difficulties, such as troubles with distance learning (e.g., lack of sufficient technology) or with evaluation of students, increased concerns about their profession, and decreased overall teacher well-being (Alves et al., 2021). Further, educators in Canada who also had the primary responsibilities of caring for family members or supporting their own child(ren)’s learning reported higher levels of depression and anxiety during this time than those who shared or did not have this responsibility (Spadafora et al., 2021). Kindergarten educators teaching in the United States through the school shutdowns that occurred during the first wave of the pandemic reported declined physical health, increased emotional stress (e.g., sadness, nervousness), and a higher proportion reported financial stress during this time (Swigonski et al., 2021). Teachers in Germany were experiencing technical barriers, as well as moderate to high levels of stress during online teaching due to school closures, though they generally reported that they were able to effectively cope with the added stress (Klapproth et al., 2020). Another Canadian study examining elementary and high school teachers during the first school shutdown of the COVID-19 pandemic (Sokal et al., 2020) found that teachers reported increased levels of burnout over the period of April–June 2020. On the positive side, teachers reported an increased sense of accomplishment in their work which the researchers attributed to their recently found ability to manage a classroom in an online environment, as these teachers also reported higher levels of efficacy during this time frame (Sokal et al., 2020).

In the first wave of the COVID-19 pandemic, the transition to online learning was sudden and thus quite challenging. Educators were forced to design and implement lessons in an online setting without any preparation or training and using only materials the children would have access to at home. Some teachers had to adapt their teaching materials for students who did not have access to a computer, the internet, or a place to complete their schoolwork (Van Lancker & Parolin, 2020). Sometimes, teachers had to prepare both online and paper versions of lessons for those in need, often resulting in increased preparation time for the teacher (Turner et al., 2020). The challenges associated with online learning may be greater for educators of younger children. Foti (2020) suggested that, due to the younger age of kindergarten students and their lack of ability to engage independently with distance learning, kindergarten educators were faced with increased challenges and difficulties, such as students being unable to use the requisite technology on their own, and the difficulty adapting their kindergarten teaching materials for distance learning. Young children require support from an adult to access online learning, log on to devices, and complete and submit homework; therefore, children who do not have an adult available to help them may face further challenges and inequities in their education. In qualitative interviews, Canadian early childhood educators discussed that their teaching practices, curriculum, and social connectedness was disrupted by the COVID-19 pandemic (Lafave et al., 2021). Moreover, research has suggested that the increased burden of responsibilities on educators had a negative impact on their mental health (e.g., Eadie et al., 2021; Spadafora et al., 2021) highlighting the importance of continuing to understand the educational context during this time.

Furthermore, not all teachers were well-positioned to implement distance learning programs when schools closed (Merrill, 2020). The sudden school closures in the first wave of the pandemic meant that even teachers who had experience with online teaching faced the additional tasks of disseminating their lessons through real-time interactive classes, pre-recorded material, and managing homework via digital classes on a full-time basis, which may not have been part of their previous curriculum. Student attendance for remote learning was also problematic. In Canada, some parents decided to withdraw their children out of distance learning altogether (Wong, 2020). Declining enrollment and attendance numbers was also an issue discussed by early childhood educators in the US throughout the pandemic (e.g., Crawford et al., 2021). Another factor that impeded the effectiveness of distance learning, particularly of primary school-aged children, was a lack of communication from parents (e.g., parents unavailable or not engaged; Timmons et al., 2021). A large study examining school-aged children from kindergarten to Grade 12 in the United States, from April to July 2020, found that when schools provided a wide range of educational options, especially live contact time with teachers, students and parents spent more time in learning activities (Bansak & Starr, 2021). In this study, the educational level of the parent was not associated with time spent helping their children, however, less educated parents tended to face more difficulties with providing resources to support their children (e.g., access to computer or internet). Additionally, children in Grades 2–6 tended to have increased behavioral issues while learning from home, including not following their parents’ directions, making it increasingly difficult to effectively conduct a lesson or have students submit assignments (Liu et al., 2021).

### Current Study

Given the difficulties associated with distance learning for teachers, students, and their families, and the potential repercussions of this disruption in children’s education, it is important to gain a better understanding of the learning environment that occurred during the school shutdowns in the spring of 2020. Data from the first COVID-19 wave suggest that school closures had a detrimental impact on children’s physical, social, and mental health (Zimmerman & Anderson, 2021). Therefore, it is particularly important that we focus our attention on the learning context of young children during these disruptions to better inform our understanding of these children’s education and development trajectories in the years to come, as the youngest learners are the ones who may experience the longest lasting impacts from this pandemic.

The purpose of the current study was to provide a snapshot of the kindergarten learning in Ontario during the first COVID-19 pandemic school shutdown in the spring of 2020. Specifically, we were interested in exploring the context of teaching from a distance and the barriers to distance learning experienced by educators, students, and their families. We expected that educators would report difficulties teaching online during the first school shutdown, such as issues with technology or inconsistent communication with parents. However, the magnitude of these barriers was unknown, as well as which barriers would be the primary obstacles, or what issues were of greatest concern to educators regarding the return to school. At the time of the study design and data collection, there was a shared belief that the pandemic would be over by the fall of 2020. Therefore, these data were also collected with the intent to inform future planning. Basic descriptive findings were shared in the summer of 2020 with stakeholders for immediate use (Janus et al., 2020). In this paper, we analysed both quantitative and qualitative responses to: (1) establish the context of learning in kindergarten during the first school shutdown of the pandemic; (2) identify the major barriers and supports to online learning; (3) provide insight on the catalysts of the potential impacts on kindergarten children’s learning trajectories; and (4) inform future preparation of teachers and educators for emergency situations such as these.

## Methods

### Study Design

The current study was a cross-sectional study of kindergarten educators during the COVID-19 pandemic-related school shutdowns in the spring of 2020. Educators in Ontario, Canada were invited to complete a survey between May and July 2020. In this multiple-method study, educators reported on their experiences using custom-created items with quantifiable, multiple answer options, validated scales, and qualitative open-ended questions. Qualitative methodology allows for a more comprehensive understanding of the viewpoints and experiences of the participant (Morse, 2012) and enables a broader exploration of unknown factors (Cypress, 2015). Given the unknowns with regards to the COVID-19 pandemic, the combination of quantitative and qualitative methods allowed participants to share their experiences, perspectives, and concerns beyond items listed in the quantitative questions.

### Participants and Procedure

In Ontario, kindergarten is a two-year program (junior and senior kindergarten) with children beginning to attend in September of the year they turn 4 years old. The mandated kindergarten curriculum is based on play-based learning (Government of Ontario, 2019a, b), with educators focused on hands-on activities to enhance student learning. The kindergarten class is taught by an educator team comprised of an early childhood educator (ECE) and a teacher (Government of Ontario, 2019a, b). Teachers are responsible for planning lessons within the provincial curriculum, assessing student learning and reporting to parents. ECEs are responsible for using their knowledge of early childhood development to plan age-appropriate hands-on activities to promote children’s overall development (Government of Ontario, 2019a, b). For the current study, participants included both types of educators from Ontario publicly-funded schools, including Anglophone and Francophone schools in both Catholic and public-school districts. Of note, in Canada, over 90% of children attend publicly-funded schools (91.8% in 2018/19; Government of Canada, 2020). Participants were asked to complete a web-based survey entitled “Hidden Future Front Line: Educators’ perspective on the impact of the COVID-19 pandemic on kindergarten children (HiFLEC)” from May to July 2020. Invitations to participate in the study were sent out through the educators’ unions. Respondents to the study included representatives from 73 out of the 74 school districts in Ontario.

The study sample consisted of participants who had a valid response for their role (i.e., either kindergarten teacher or ECE) and on at least one of the study variables (*n* = 2569). Kindergarten teachers comprised 74.2% (*n* = 1906) of the sample. They were predominantly female (97.6%), were mostly 36 years of age or older (80.6%), and almost three quarters of the sample reported being a teacher (72.1%) or ECE (72%) for more than 10 years. Participation in the study was voluntary and all procedures received ethical clearance from the University’s Research Ethics Board.

### Measures

#### Demographics

Educators reported on their personal demographics (e.g., age bracket, sex, years of experience), as well as the demographics of their current classroom (e.g., location of school).

#### Experiences Related to COVID-19

Participants reported on their teaching arrangements at the time of the school shutdown, barriers to distance learning, and their connections with students and their families. Open-ended questions were included to allow participants to expand on their feelings and experiences with regards to issues parents contacted them about, barriers to learning, and concerns for returning to school. Participants also completed the COVID-19 Family Experiences Scale, a set of questions on their current home life as it pertained to the COVID-19 pandemic, adapted from items used in concurrent pandemic-related research across Canada (Khoury et al., 2021).

### Analytical Strategy

Descriptive statistics, including means, proportions, and frequencies, were used to examine educator responses to the quantitative questions. All statistics were conducted using SPSS version 25 (IBM Corp., 2017). Open-ended responses were analyzed by two independent coders. First, coders went through unique sets of responses (in both English and French) to apply first-level codes. Next, the coders collaboratively reviewed the codes and first-level codes were further refined (Charmaz, 2006). Any discrepancies were resolved through a discussion and consensus among the two coders. Responses were grouped together by theme and a third independent coder spot checked coding for each question. Two of the three coders were fully bilingual in English and French. Given the large number of responses, there were several themes for each question. We have therefore presented the most salient themes in our results section and listed all themes in Supplemental Table S1.

## Results

### School and Class Characteristics

Educators reported on their school location and the children in their class. Just over a third of educators reported their school was located either in an urban (32.5%; *n* = 835) or suburban area (31.8%; *n* = 818), while 13% (*n* = 335) reported their school was in a rural area; 10.8% (*n* = 277) reported a semi-urban location, and 8.1% (*n* = 208) a mixed one, with 2.5% (*n* = 65) saying they didn’t know.

**Fig. 1:**
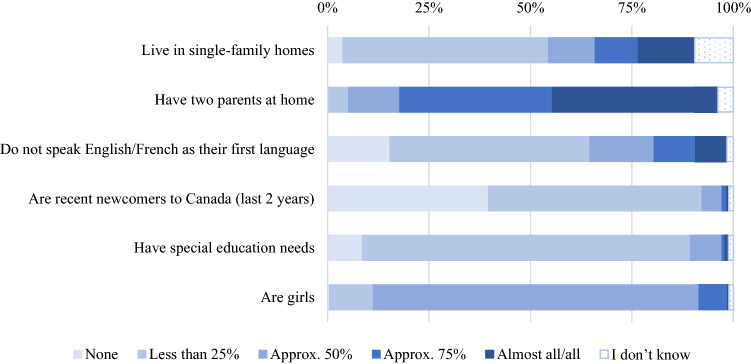
Percentages of children in demographic categories reported by educators

Class composition characteristics are shown in Fig. 1. About half (*n* = 1288) of all educators reported that less than 25% of their students lived in single-family homes, while 40% of educators (*n* = 1040) reported that all or almost all their students had two parents at home. Roughly half of the educators (*n* = 1257) also reported less than 25% of their students’ first language was something other than the language of instruction (either English or French) and were newcomers to Canada (*n* = 1281). Most educators (81%, *n* = 2065) reported fewer than 25% of their students had a special educational need and 80% (*n* = 2049) reported approximately half their students were girls.

### The Context of Distance Learning in Kindergarten

**Fig. 2:**
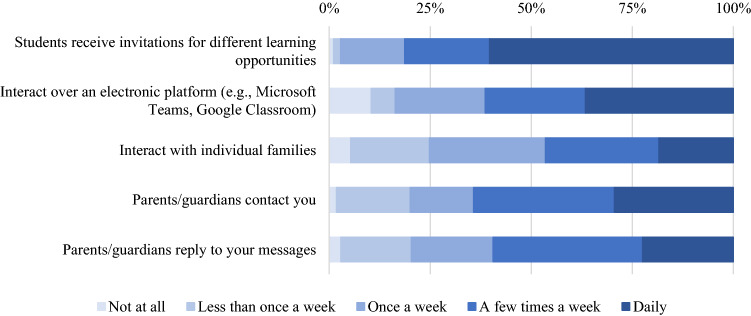
Percentages of reported learning arrangements during the first COVID-19 school shutdown

Figure 2 provides specific information regarding the online learning in kindergarten classrooms. Fifty-eight percent of respondents (*n* = 1495) reported that students in their class received invitations for different learning opportunities every day, however only 35.9% (*n* = 923) reported that they had daily online interactions with their class. The majority of educators also reported weekly communication with individual families: 78.8% (*n* = 2022) reported parents contacted them a least once a week, 73.4% (*n* = 1886) reported interacting with individual families at least once a week, and 70% (*n* = 1793) reported that parents responded to their messages at least once a week.

More than half (51.5%; *n* = 1322) agreed or strongly agreed they had the necessary resources, while 26.6% (*n* = 683) disagreed, and 21.6% (*n* = 554) neither agreed nor disagreed. A minority (39%; *n* = 1001) of the kindergarten educators felt that they were able to provide individualized learning opportunities to students of different levels. With regards to time spent preparing for class each week, only 22.5% (*n* = 577) stated that they were spending the same amount of time or less preparing for class, whereas 76.2% (*n* = 1957) stated they were spending more time developing their lessons each week. Over half (56%; *n* = 1439) of the educators surveyed reported that their online interactions with their class were 30 min or less.

#### Connections with Families

**Fig. 3:**
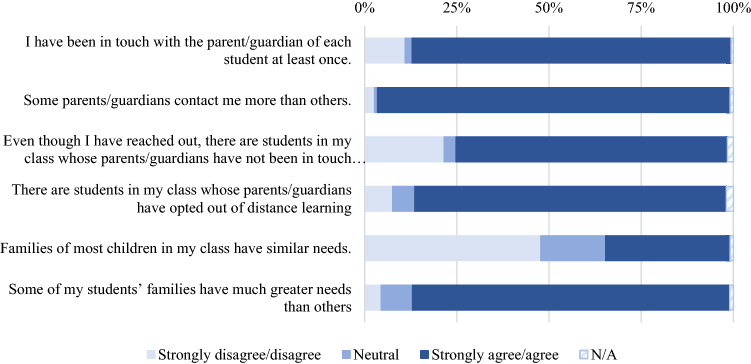
Percentages of communication and connection types between educators and their students’ families

A total of 86.5% (*n* = 2051) of educators stated that they had been in touch with a parent or guardian of each student at least once since the beginning of the school closures. However, 73.7% (*n* = 1745) reported that there were parents/guardians of students in their class that had not been in touch with them since the schools closed. The majority of the sample (78%, *n* = 2005) stated that they had at least one student in their class whose parents or guardians had opted out of distance learning. Figure 3 displays the percentages of educator responses for each statement regarding connections with student families.

#### Reasons Why Parents Contacted Educators

**Fig. 4:**
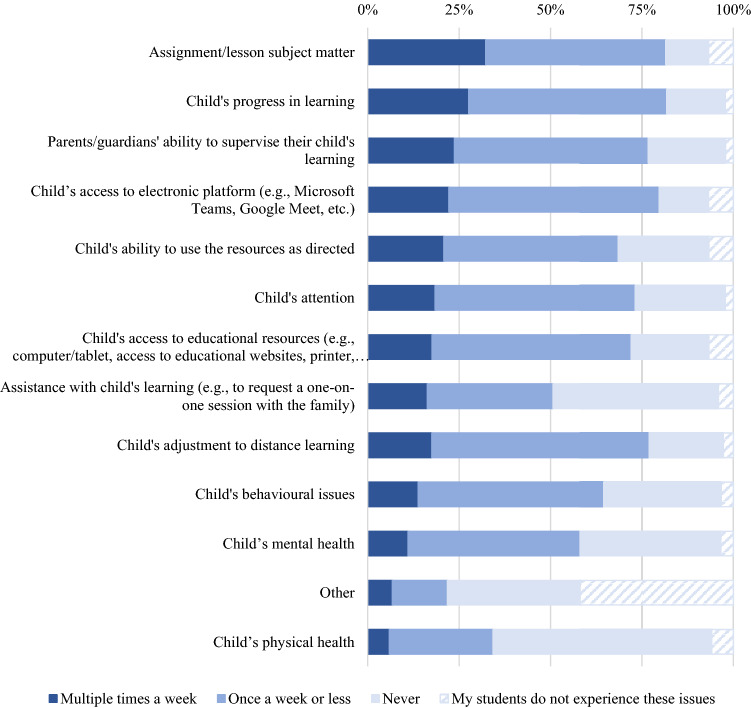
Educator-reported rates of how often parents/guardians contacted them about certain issues

Educators reported how often parents contacted them regarding a variety of issues (Fig. 4). The highest reported reason parents contacted educators was regarding assignment/lesson subject matter (29.4% multiple times a week, *n* = 756), while the least reported reason was about the child’s physical health (5.4% multiple times a week, *n* = 137).

Educators who indicated that there were other issues that parents/guardians contacted them about were presented with the following open-ended question: “What other issues do parents/guardians contact you about?” The largest theme reported by educators was technological concerns. This included not being able to access the necessary platforms, glitches in the systems, or issues with internet connections:

I have a few families that seem to have endless trouble with passwords, using technology, experiencing glitches & needed tech support. I have spent hour [*sic*] and hours helping a few of them

Limited wifi connection.

Parents need assistance with technical issues and ask for assistance to open documents, download activities, login to Dreambox, RazKids, Splash Math (forgotten passwords), upload photos of their child’s work, troubleshoot problems with their devices

The second largest theme educators reported regarding reasons why parents contacted them was related to them not being able to support or teach their child effectively or the inability to engage them in their work, as evidenced by the examples below:

How to teach letters, vowels etc., pedagogical questions

Ability to teach their kids. Time needed to teach them Not knowing how to support when kids struggle. Not knowing how to persist when child lacks interest or skills. Not knowing how long to work at a task. Not knowing how to adjust task to child’s needs

How to teach them a specific skill. Or asking if they are teaching something correctly.

The next most common theme was concerning parents contacting educators just to check in or to report on their child’s activities, as seen below:

Sharing other learning that they did at home with their children.

The parents who engage often like to send texts and photos of their children’s everyday activities which they usually have apologized for as ‘not part of the learning’

Some parents just want to chat and check in.

Lastly, educators reported that many parents/guardians contacted them about family issues or instability occurring at that time:

One family needed to discuss sharing their child’s custody back and forth, and how they were trying to assist their child in each environment...

One parent called to explain that her husband and my student/her child had COVID-19, both recovered.

I have had many parents ask for financial assistance. I had a parent pass away during covid so I was communicating with the family to help support. I had a mom contact me because she had surgery and had no support…

Along similar lines, among the comments made by educators was a theme relating to stress due to the COVID-19 pandemic and the associated shutdowns:

Many parents had a difficult time keeping things professional and when phone calls were made, they often wanted to discuss all of the problems going on in their lives, instead of just the things related to their child and education...

One child was very upset when he saw people wearing masks while grocery shopping. I think they just don’t know what to make of all this.

Just them telling us they are tired and exhausted from all of this

### Barriers to Distance Learning

The survey also asked educators about any barriers to distance learning they or their students may have experienced. Among barriers experienced by students and their families, the number one barrier to effective teaching, reported by 88% (*n* = 2262) of respondents, was parents/guardians not submitting assignments or providing updates on their child’s learning. Next, over three quarters of the sample (77%; *n* = 1980) reported diverse student needs being a barrier to effective teaching. About half of the educators (*n* = 1335) reported that a lack of reliable internet at home was an issue and that students’ lack of access to electronic devices (*n* = 1274) was also an issue (despite 77%; *n* = 1979) of respondents indicating that at least one student in their classroom received an electronic device from the school district). Lastly, 48% (*n* = 1232) reported privacy concerns around distance learning.

**Fig. 5:**
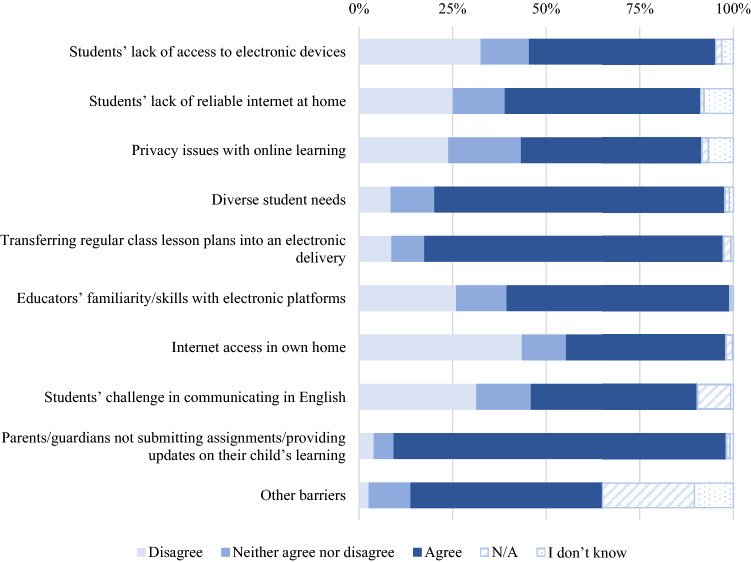
Percentages of reported barriers to effective teaching during online learning due to the COVID-19 pandemic-related school closures

Among barriers they experienced themselves, the majority of the educators (79.5%, *n* = 2041) reported difficulty modifying their lesson plans to be suitable for online delivery. Almost two thirds of educators (59.1%, *n* = 1519) stated they felt their own lack of familiarity with the electronic platforms they were using hindered distance learning, and 42% (*n* = 1085) of educators reported issues with internet access in their home. A full breakdown of responses to barriers to distance teaching are presented in Fig. 5 (strongly (dis)agree and (dis)agree responses were combined for ease of presentation).

Educators who selected that they experienced other barriers to distance learning had once again the opportunity to describe these barriers in an open-ended field text box. Educators were asked: “What are the other barriers to distance learning you may have experienced?” The barrier reported most often by educators was that their students’ parents/guardians had to balance work (either from home or outside the home) and their responsibilities with their child’s learning:

Parents are busy working, even if they are working from home. With this age group they have to all [*sic*] the work. If they have multiple children it is exceedingly difficult…

Parents who had to continue working through the entire pandemic. It was hard for them to help their children participate as they got home at 5-6 pm. Their children were tired and not interested in participating at that time.

Parents having to manage distance learning while having to care for multiple younger Children including toddlers and infants.

Educators also discussed difficulties balancing online teaching with caring for and supporting their own children’s learning:

Trying to balance teaching and taking care of and supporting learning for my own children full time.

Being able to be connected when my own children are at home and needed to be connected; helping my children get their learning done during the day;

I have two children at home that I am also teaching. This caused problems especially when I needed to plan and respond to parents regularly. Most nights I was up to midnight answering parent emails

The next theme reported by educators was the lack of independence of kindergarten students. Specifically, they reported how kindergarten-aged students are unable to log on and engage with any distance learning without the support of someone at home:

These kids can’t read and cannot independently access these lessons, therefore participation falls on the parent not the child, which is not really the case in older grades.

The kindergarten children usually need 1:1 support to complete school activities. Once they are logged onto the Google Classroom, some of the children can navigate the interface, but they need help to gather the manipulatives (e.g. beans, cutlery, dice) and usually guidance to complete the task provided.

Kinders need parent support for pretty much 100% of the activities I provide.

Kindergarten educators also reported the lack of ability to truly carry out the Ontario play-based curriculum in an online setting:

Teaching kindergarten is a hands-on experience. It is difficult to teach students reading and writing when they are just beginning to learn letter sounds and basic sight words. We need to be there with them to give decent instructional time. This can’t be achieved over a computer or ipad.

Lack of opportunity to develop social, emotional, developmental skills when not with their peers, and cannot be ‘taught’ like literacy or numeracy—lack of opportunity for the Play Based Learning^1^ that is expected practice in schools.

This is the complete opposite of what the Full Day Play Based Learning1 is all about. Children need to manipulate with concrete objects, plan and investigate during play, interact with their peers and not swipe across a screen.

### Concerns About the Return to School

**Fig. 6:**
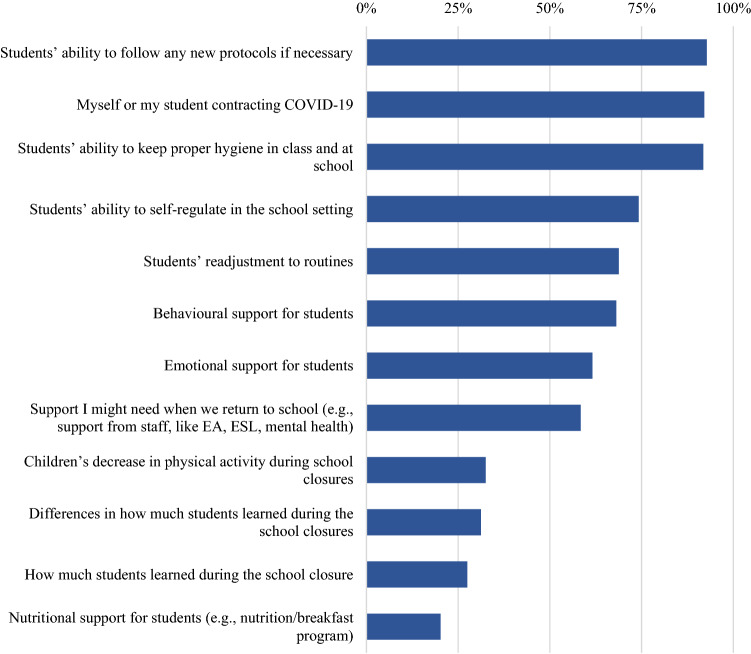
Percent educators’ reporting concerns regarding returning to school in September 2020

Educators were also asked whether they were concerned about returning to in-person teaching in September 2020, and if so, why. The majority of educators (77.2%, *n* = 1982) stated they were concerned about the return to school. Of those educators, when asked what they were concerned about, the most frequently reported educator concerns were the ability of kindergarten students to follow any new protocols and keep proper hygiene while at school (70.8%, *n* = 1819), and the fear of students or themselves contracting the virus (71%, *n* = 1825). See Fig. 6.

Following this, educators had the opportunity to respond to the open-ended question of, “I am also concerned about:” The largest theme reported by educators was their concerns around the ability of their young children to engage in social distancing protocols, as demonstrated below:

Children not being able to socially distance; not understanding the protocols to help with minimizing the spread of COVID and other illnesses.

I am also concerned with the ability to maintain social distancing regulations that I have heard were implemented in other countries when schools reopened. Young children struggle to understand the need to distance from others.

How realistic is it to expect students to ‘socially distance’ in kindergarten?

Along similar lines, educators discussed once again the lack of independence of kindergarten students, highlighting their inability to properly assist their students while being physically distanced:

Being a Kindergarten teacher is a very physical job where we are in physical contact with students every day, zipping up coats, helping with shoes, changing a child in the bathroom after an accident.

Many students come in not knowing how to toilet, zip a zipper or get on their shoes. Educators assist students with so many everyday activities and are in physical contact.

Who’s supporting all these students in the bathroom? I could go on and on!

Educators also discussed cleaning/sanitizing issues, concerned with not only the overall classroom environment, but also the sharing of resources and manipulatives in kindergarten classrooms:

I am concerned about what materials/toys we will be allowed to use and how and who will clean them.

Who’s responsible for cleaning our room? How do we know it’s being done because I can tell you that it wasn’t being cleaned before all this other then by the educators in the room!

There aren’t enough washrooms for the school, staff, or in my room. Who’s sanitizing? Toys? What’s allowed? Who’s cleaning and sanitizing? Desks? We only have tables.

Lastly, educators discussed their fear of contracting the virus while at work and passing it on to their loved ones:

The fact that I have no idea who they have been in contact with or their parents and if they are respecting the 2 m rule. The fact that at home my husband is battling cancer and that I myself can not and will not put myself in a position of being surrounded by 30 kindie kids!

I’m concerned about returning too early and therefore many people contracting the virus.

What/who/how large of circle students have been exposed to and potential risk of bringing covid19 to the classroom

## Discussion

The current study offered a snapshot of the learning situation of educators in the virtual kindergarten classes during the COVID-19 pandemic-related school shutdowns that occurred in Ontario in the spring of 2020. Educators reported that parents contacted them most often about technological issues or how to effectively support their child in their learning. The most frequently reported barriers to distance learning included balancing work, life, and online learning (for both parents and educators), the lack of independence of kindergarten children, and their uncertainty regarding how to carry out the play-based Ontario kindergarten curriculum. With regards to the return to school in the fall of 2020, educators reported that they were concerned about children’s ability to follow safety protocols and how to support children while maintaining physical distance. Overall, our study provides information about the context of learning during the first school shutdown due to the COVID-19 pandemic, which will contribute to our understanding of the short- and long-term implications of pandemic-related shutdowns on children’s development and education. Educators’ comments on the unique challenges of distance learning among the youngest students also provided an affirmation of our choice to focus our research specifically on this population.

More than three quarters of educators reported they were spending more time preparing for classes each week compared to before the pandemic, though over half of the sample reported that their daily interactions with their class were thirty minutes or less. Based on the qualitative responses, the increased preparation time seemed to be due to the challenges of planning online lessons and activities appropriate for kindergarten-aged children that could be done effectively at home. These findings are consistent with other pandemic research that emphasized the burden and unsustainability of adapting elementary school lessons to an online medium (Turner et al., 2020).

The largest reported barriers in both the quantitative and qualitative responses were focused on parental involvement and engagement with regards to kindergarten children’s learning (e.g., not submitting work or too busy to help children due to their employment). Many parents were unable to support their children as they were either working from home or outside of the house, and unable to provide their young children with the consistent support required to be successful in a virtual kindergarten classroom. Educators also mentioned that many parents were stressed due to pandemic-related circumstances, and often unable to provide the necessary support for their children or chose not to participate in distance learning at all. Indeed, other research exploring the context of kindergarten in Canada during the COVID-19 pandemic found that parental involvement levels were positively associated with their children’s academic achievement, and that parental involvement in their child’s learning greatly varied, with some parents able to support their child daily, while other parents were only able to help as their own time permitted (Timmons et al., 2021). Literature to date has established that children’s skills in kindergarten are predictive of their future academic achievement (e.g., Goldstein et al., 2017), therefore making it especially important for us to understand the learning that occurred in kindergarten as it has the potential to impact later academic development.

Two other barriers to distance learning, as reported by educators in both their quantitative and qualitative responses, were related to issues with technology and the lack of electronic devices. Educators discussed families not having enough devices for all family members and prioritizing the older children in the family, the lack of adequate internet connections, and even privacy concerns (e.g., lack of dedicated space in their home for teaching). Educators in China reported similar issues with distance learning, such as the lack of self-regulation of young children, ineffectiveness of online teaching, and parents being unprepared to support the online learning of their children (Dong et al., 2020). Elementary school teachers in Indonesia also reported difficulties modifying their instructional strategies using online platforms, a lack of student access to devices, and decreased student engagement in a virtual setting (Aliyyah et al., 2020).

A unique challenge reported by the educators in our study was the lack of ability to effectively carry out the play-based early learning curriculum mandated in Ontario (Government of Ontario, 2019a, b). The current early learning curriculum is focused on inquiry-based learning through play and social interactions and as discussed by the educators in our study, this is nearly impossible to effectively carry out through remote learning. In addition, another curriculum-related concern expressed by educators was related to the cleaning and sanitization of shared toys or class materials (crayons, markers, blocs, etc.) for learning within a kindergarten classroom setting. They also not only mentioned concerns regarding the anticipated challenge of keeping young students physically distant from one another when the typical kindergarten curriculum encourages them to interact with each other and share materials, but also expressed concerns about how to effectively carry out kindergarten lessons while adhering to all the pandemic-related public health measures. These findings suggest that perhaps special considerations, such as how to effectively support the social interactions of young children, may need to be made to ensure the kindergarten curriculum can be effectively carried out during potential school closures.

An additional barrier to distance learning noted by educators was balancing their own family responsibilities during the COVID-19 pandemic while having to teach from home. Since all schools and childcare centers were closed during this time, educators who were also parents had the added responsibility of having to care for or support their own children with their schooling. Our previous research has found that this added responsibility was negatively associated with educator mental health (Author-blinded for peer review). The qualitative comments in the current study provided further insight into educators’ specific feelings and concerns about balancing work and home responsibilities. For example, educators who were also parents noted the difficulty of balancing their time and responsibilities during the day. This theme is further supported by a recent study that found that, during the pandemic, educational professionals who had young children of their own to care for had higher levels of stress related to their distance teaching, compared to their counterparts who did not have this responsibility (Košir et al., 2020).

A theme relatively unique to the online learning of the youngest learners is their lack of independence. For instance, not only are kindergarten children unable to access work or engage in virtual interactions without the support of an adult, as mentioned above, they also need adult support to complete school-related tasks, read assignment instructions, and upload assignments. Young children’s lack of independence impedes their ability to engage with remote learning without the support of an adult (e.g., Foti, 2020). The notion of independence was also discussed when educators were asked about their concerns about returning to school. Educators were concerned about the inability of 4- to 6-year-old children to physically distance or adhere to potential health and safety protocols that may be in place. For example, kindergarten-aged students often need support with daily tasks such as zipping up their coats or opening their lunch containers—tasks that are difficult to help with while standing six feet away. Educators were concerned how to effectively teach kindergarten students while also following public health guidelines that may be in place.

### Strengths, Limitations and Future Direction

Our study provides important insight into what kindergarten learning looked like in Ontario during the first COVID-19 pandemic-related school closures, however, there were some limitations that should be acknowledged. While a strength of our study is that we had representation from 99% of school districts/authorities in Ontario, our study is only representative of the play-based kindergarten program in Ontario (Government of Ontario, 2019a, b). Education is a provincial mandate in Canada, therefore the kindergarten curriculum and program vary by jurisdiction—some jurisdictions have full-day kindergarten, while others only have half-days or full-day on alternating days. Unlike Ontario, most provinces only have one year of kindergarten and not two. The components of play-based learning are also included to varying degree. Our study provides information on distance learning in Ontario and can be compared with potential reports from other regions to understand children’s development across provinces. Research should continue to report on pandemic-related issues in schooling in both Ontario and across all the Canadian provinces, as well as across all grade levels.

Our data were collected from May to July 2020 and therefore only provide insight into kindergarten learning during the first COVID-19 pandemic-related school closures. The teaching and learning that occurred during the first school shutdown was quite different from the learning that occurred in person or during later school closures. During the first set of school closures, educators and school boards were not adequately prepared to move to online platforms and firm guidelines about the amount and type of daily instruction were not yet in place. Following this first shutdown, the provincial government did lay out guidelines for the type and amount of instruction for both in person and virtual learning for the return to school in the fall of 2020. However, the findings of the current study are important to report and discuss, as these experiences contributed to preparation for later shutdowns and informed future insight into the initial shock of what happened when the pandemic began. Future research should explore kindergarten when students returned to class in the fall, and also during subsequent school closures. We expect that the educational trajectories of children whose learning was disrupted by theandemicc will be monitored closely in the future, and thus our study will contribute to further understanding of the possible impact of the disruptions on the youngest learners. There appears to be an emerging consensus that school closures should be a measure of last resort in fighting a pandemic (Buonsenso et al., 2021), which has the potential to mitigate any further learning losses.

### Conclusion

Previous research suggests that children have been and will continue to be negatively impacted by the pandemic-related school closures, both psychosocially (Phelps & Sperry, 2020) and academically (e.g., Engzell et al., 2021). It is therefore important that we continue to gather information on the context of learning that occurred during these closures. Our study highlights the unique issues that parents and educators of kindergarten-aged children in Ontario dealt with during the first set of school closures in the spring of 2020. It will be many years before our society will fully understand the magnitude of the COVID-19 pandemic-related school shutdowns, however, these preliminary studies, particularly examining the context of learning of the youngest students, will be vital in our continued understanding of the impact of the COVID-19 pandemic on children.

## Supplementary Information

Below is the link to the electronic supplementary material.

Supplementary file1 (DOCX 16 KB)

## Data Availability

Due to REB regulations, data are confidential and can not be shared.

## Footnotes

1 The educator refers to the Ontario kindergarten curriculum: The Ontario Curriculum: Elementary. (Government of Ontario, 2019a, b).
