# Supplementary material for: From Full Day Learning to 30 Minutes a Day: A Descriptive Study of Early Learning During the First COVID-19 Pandemic School Shutdown in Ontario
Source: Early Child Educ J. 2022 Jan 16;51(2):287–99. doi: 10.1007/s10643-021-01304-z (PMC8761377; doi:10.1007/s10643-021-01304-z)
Supplement: Supplementary file 1 — Supplementary file1 (DOCX 16 KB) [file 10643_2021_1304_MOESM1_ESM.docx]

**Table S1.**

*Full list of qualitative themes derived from the open-ended response options on the HiFLEC survey*

| **Open-ended question** | **Full list of themes** |
| --- | --- |
| Reasons Parents Contacted Educators About | Technological concerns  Retrieval of supplies at school  Mental health (of parent or child)  Ability to support/engage child to learn  Concerns about returning to in new school year/transition to grade 1  Checking in with teacher or reporting child’s activities/progress  Inability to engage in learning due to balancing other demands (e.g., work and learning of multiple siblings)  Family issues/instability  General anxiety/frustration around current situation (e.g., COVID-19, school closures)  Concerns around lack of social interaction/missing friends  Concerns or questions about child assignments (e.g., not hard enough, wanting additional work)  Gratitude (i.e., parents contacting to say thank-you)  Language Barriers (e.g., for French classes, no one at home who speaks French) |
| Barriers to Distance Learning | Parents balancing work/child learning/home life responsibilities  Lack of independence of kindergarten students  Issues with or lack of access to technology  Parental lack of ability or knowledge to support child  Parent lack of ability or knowledge with technology  Lack of participation of families  Lack of communication with families  Educators balancing online teaching with home life responsibilities  Inability to assess child effectively/parents doing work for child  Difficulty adapting play-based learning/social interaction pieces of kindergarten to an online format  Language barriers (either ESL families or not having a French speaking parent for French immersion students)  Lack of resources/materials of families  Lack of student/family engagement  Parents not seeing value in kindergarten education  Multiple children in online learning older students taking priority/having to share devices  Lack of support for educators/mixed guidelines from ministry  Children in care of someone else during the day (e.g., grandparents) or not having environment conducive to learning in home  Family issues/parents feeling overwhelmed  Increased screen time of children  Lack of support for children with special needs |
| Concerns About the Return to School | Inability to social distance  Risk of virus exposure and spreading to own family  Sick kids coming to school  Mental health/well-being (of students and teachers)  School adjustment/separation anxiety when starting school again  Fear of unknown  Lack of information/guidelines from the boards and government  Potential for hybrid or staggered learning  Lack of support for special needs students  Ability to comfort/support students from a distance  Lack of social-emotional development (e.g., due to lack of interaction, wearing masks)  Issues with Staff  Lack of independence of young students (need to be helped within physical distance)  Personal protection equipment concerns  Funding for added costs/lack of resources  Cleaning/sanitizing issues (including sharing physical resources)  Class sizes  Sick leave protocols  How to effectively teach play-based kindergarten in the new environment  Lack of hygiene of young children  Regression due to lack of learning during lockdown  Parents’ anxiety surrounding sending kids to school  Ability to enforce COVID-19 protocols |
